# GCH1-polymorphism and pain sensitivity among women with provoked vestibulodynia

**DOI:** 10.1186/1744-8069-8-68

**Published:** 2012-09-12

**Authors:** Ulrika Heddini, Nina Bohm-Starke, Alfhild Grönbladh, Fred Nyberg, Kent W Nilsson, Ulrika Johannesson

**Affiliations:** 1 Department of Clinical Sciences, Danderyd Hospital, Karolinska Institutet, Division of Obstetrics and Gynecology, Stockholm, Sweden; 2 Department of Pharmaceutical Biosciences, Division of Biological Research on Drug Dependence, Uppsala University, Uppsala, Sweden; 3 Center for clinical research, Uppsala University, County Council of Västmanland Central Hospital, Västerås, Sweden

**Keywords:** Provoked vestibulodynia, Pain, GCH1, Gene, Polymorphism, SNP, Hormonal contraceptives

## Abstract

**Background:**

Provoked vestibulodynia (PVD) is a pain disorder localized in the vestibular mucosa. It is the most common cause of dyspareunia among young women and it is associated with general pain hypersensitivity and other chronic pain conditions. Polymorphism in the guanosine triphosphate cyclohydrolase (*GCH1*) gene has been found to influence general pain sensitivity and the risk of developing a longstanding pain condition. The aim of this study was to investigate *GCH1*-polymorphism in women with PVD and healthy controls, in correlation to pain sensitivity.

**Results:**

We found no correlation between the previously defined pain-protective *GCH1-*SNP combination and the diagnosis of PVD. Nor any correlation with pain sensitivity measured as pressure pain thresholds on the arm, leg and in the vestibule, coital pain scored on a visual analog scale and prevalence of other bodily pain conditions among women with PVD (n = 98) and healthy controls (n = 102). However, among patients with current treatment (n = 36), there was a significant interaction effect of *GCH1*-gene polymorphism and hormonal contraceptive (HC) therapy on coital pain (p = 0.04) as well as on pressure pain thresholds on the arm (p = 0.04). PVD patients carrying the specified SNP combination and using HCs had higher pain sensitivity compared to non-carriers. In non-HC-users, carriers had lower pain sensitivity.

**Conclusions:**

The results of this study gave no support to the hypothesis that polymorphism in the *GCH1*-gene contributes to the etiology of PVD. However, among patients currently receiving treatment an interaction effect of the defined SNP combination and use of hormonal contraceptives on pain sensitivity was found. This finding offers a possible explanation to the clinically known fact that some PVD patients improve after cessation of hormonal contraceptives, indicating that PVD patients carrying the defined SNP combination of *GCH1* would benefit from this intervention.

## Background

Provoked vestibulodynia (PVD) is the most common cause for superficial dyspareunia in young women, with a prevalence of 13-15% [1]. PVD is a localized pain disorder [2] characterized by pain provoked by touch, pressure and stretch of the tissue around the vaginal opening often resulting in inability to engage in vaginal intercourse. The condition can be divided into primary PVD, defined as pain ever since first tampon use or vaginal intercourse and secondary PVD, where dyspareunia appears after a period of pain-free sexual intercourse [3]. The etiology of PVD is still unclear and both biomedical and psychosexual triggers are being discussed [4-9].

The pain mechanisms involved in PVD are not fully understood. Studies have shown an increase of free nerve endings, immuno-positive for the neurotransmitters calcitonin gene-related peptide (CGRP) and substance P (SP), as well as an increased local blood flow in the mucosa around the vaginal opening [10-12]. The nerves are of sensory origin and are sensitized with low pain thresholds for most stimuli suggesting a chronic neurogenic inflammation [13,14]. There is also evidence of involvement of central pain mechanisms. Women with PVD have lower pain thresholds also in other body areas and more concomitant bodily pain compared to controls [15-17]. In addition, correlations to other pain conditions such as fibromyalgia and irritable bowel syndrome have been reported [4,18].

A familial aggregation of PVD has not been proved, although it has been found in other pain syndromes associated with PVD [19,20]. A limited number of studies have investigated a possible genetic predisposition to develop PVD, focusing on polymorphism in genes involved in the pro-inflammatory immune-response [21-25]. There are an increasing number of studies on genetic polymorphism influencing endogenous pain modulation but this has not been studied among women with PVD. Tegeder and colleagues have reported that specific single nucleotide polymorphisms (SNPás) in the guanosine triphosphate cyclohydrolase (*GCH1*) gene are associated with reduced pain sensitivity in humans [26]. *GCH1* is the rate limiting enzyme in the biosynthesis of 6(R)-L-erythro-5,6,7,8-tetrahydrobiopterin (BH4). BH4 is an essential cofactor in the synthesis of several pain modulators including catecholamines, serotonin and nitric oxide. BH4 regulates the activity of *GCH1* via feed-forward activation of phenylalanine and feedback inhibition. The identified pain-protective SNP combination of *GCH1* is composed of 15 SNPás found at different locations on the gene. Screening for three of these SNPás has been shown to be a reliable way to identify the pain-protective SNP combination with high sensitivity and specificity [27]. Subsequently, several studies have linked *GCH1*-polymorphism to various aspects of pain including neuropathic and inflammatory pain, whereas others have not [28-35]. A recent study investigating a possible association between different SNP combinations in the *GCH1*-gene and a number of pain behavior related outcomes during labor indicated a very limited effect [36].

Many pain conditions, such as tension headache and fibromyalgia are more prevalent in women than in men. The possible effect of sex hormones on pain sensitivity has been investigated in several studies [37,38]. There is evidence suggesting that estrogens influence pain modulation, however, both pro- and anti-nociceptive effects have been found. For some stimuli, lower pain sensitivity has been found during the follicular phase compared to the luteal phase in normally menstruating women, although inconsistent results exists [39-41].

In this study we aimed to investigate whether polymorphism in the *GCH1-*gene is related to PVD, hypothesizing that the pain-protective SNP combination previously identified would be less frequent among women with PVD. Another aim was to explore possible correlations between *GCH1*-polymorphism and vestibular and general pain sensitivity in women with PVD and in healthy controls, including analyzes of a possible interaction between the studied SNP combination and use of hormonal contraceptives (HCs).

## Results

### Clinical data

Clinical data of participants are shown in Table 1. In the patient group, 60 participants had completed their treatment and 38 were currently receiving treatment for PVD. For participants who had completed their treatment the median time since completion was 5 years (range 2 months-11 years). Among patients with current treatment 41% were using HCs and among those with completed treatment 30%, with no significant difference.

**Table 1:** Clinical data

**Variables**	**Patients**	**Controls**	**p-value**
	**(n = 98)**	**(n = 102)**	
Current age, years	29 (19–44)	24 (18–35)	<0.001
Duration of PVD, years	8 (0,5-18)	-	-
Primary PVD	35 (36%)	-	-
Secondary PVD	63 (64%)	-	-
Current use of hormonal contraceptives	33 (34%)	53 (52%)	0.005
Combined hormonal contraceptives	26 (27%)	42 (40%)	ns
Progestogen only contraceptives	7 (7%)	11 (11%)	ns
Menstrual cycle day	7.9 (4–13)	8.0 (3–13)	ns
**Concomitant pain**			
Dysmenorrhea	67 (71%)	55 (54%)	0.02
Headache	59 (60%)	30 (29%)	<0.001
GI pain and dysfunction	53 (54%)	22 (22%)	<0.001
Back pain	48 (49%)	20 (20%)	<0.001
Muscle pain	31 (32%)	2 (2%)	<0.001
Other pain	26 (27%)	1 (1%)	<0.001

PVD = provoked vestibulodynia, GI = gastro-intestinal.

### Genotyping

Genotyping for the studied SNP combination of *GCH1*-gene was completed in 200 subjects; 98 patients and 102 controls. The SNP frequencies in the total sample were: dbSNP rs8007267G > A: GG = 134 (67%), AG 60 (30%) and AA = 6 (3%); dbSNP rs3783641A > T: AA = 129 (64.5%), AT = 62 (31%) and TT = 9 (4.5%); dbSNP rs10483639C > G: CC 129 (65%), CG + GC 61 (31%) and GG 9 (5%). The frequencies of the different SNPs were in accordance with the Hardy-Weinberg equilibrium (dbSNP rs8007267G > A: *χ*^2^ = 0.03, p = 0.870; dbSNP rs3783641A > T: *χ*^2^ = 0.1, p = 0.755; dbSNP rs10483639C > G: *χ*^2^ = 0.09, p = 0.762).

Individuals were classified as homozygous, heterozygous or non-carriers of the specified SNP combination according to Lötsch et al. [27]. The frequencies of the SNP combinations are shown in Table 2. 

**Table 2:** **Carrier frequencies of the specified SNP combination of*GCH1***

	**Non carriers**	**Homozygos carriers**	**Heterozygos carriers**
All participants (n = 200)	139 (70%)	5 (2%)	56 (28%)
Patients (n = 98)	70 (71%)	3 (3%)	25 (26%)
- current treatment (n = 38)	28 (73%)	1 (3%)	9 (24%)
-completed treatment (n = 60)	42 (70%)	2 (3%)	16 (27%)
Controls (n = 102)	69 (68%)	2 (2%)	31 (30%)

### SNP combinations in relation to PVD

There were no differences in the SNP frequency among the patients (current and completed treatment together or separated) and controls. Nor were there any differences in SNP carrier frequency between patients with primary or secondary PVD.

### Pain measurements

There were significantly higher pressure pain thresholds (PPTs) on the arm and leg among controls compared to patients with current treatment as well as to the patient group as a whole (p = 0.002). Moreover, controls had lower self-reported bodily pain scores. Among patients with current treatment there were significantly higher scores in coital VAS pain and lower PPTs in the vestibular area B compared to patients with completed treatment. See Table 3.

**Table 3:** Pain measurements

	**Current treatment (n = 38)**		**Completed treatment (n = 60)**		**Controls (n = 102)**		**P-value**
	**Mean (SD)**	**Median (Q1-Q3)**	**Mean (SD)**	**Median (Q1-Q3)**	**Mean (SD)**	**Median (Q1-Q3)**	
**PPT leg (kPa)**	389 (156)	377 (279–454)	417 (164)	403 (316–513)	475 (152)	457 (363–575)	0.006 (ANOVA), 0.004 (K-W)
**PPT arm (kPa)**	249 (102)	215 (190–288)	279 (136)	248 (188–335)	309 (116)	298 (227–355)	0.028 (ANOVA), 0.004 (K-W)
**PPT vestibulum A (g)**	43 (28)	30 (23–60)	51 (33)	46 (25–64)	-	-	ns
**PPT vestibulum B (g)**	27 (19)	20 (13–38)	51 (53)	34 (20–70)	-	-	<0.002 (*t*-test), <0.05 (M-WU)
**Coital VAS pain (0–100)**	71 (22)	73 (56–88)	40 (31)	28 (17–68)	-	-	<0.001 (*t*-test), <0.05 (M-WU)
**Bodily pain score (0–5)**	2.1 (1.3)	2 (1–3)	2.1 (1.2)	2 (1–3)	0.8 (0.9)	1 (0–1)	<0.001 (ANOVA), <0.001 (K-W)

PPT = Pressure pain threshold, kPa = kilopascal, A = anterior vestibule, B = posterior vestibule, VAS = visual analogue scale, K-W = Kruskal-Wallis test, M-WU = Mann–Whitney *U*-test.

There were no significant differences in PPTs on the arm or leg between patients, controls or all participants together using HCs or not. Among patients there were no differences in vestibular PPTs, coital VAS pain or bodily pain score between users and non-users of HCs. Nor when users of combined or progestogen only HCs were analyzed separately.

### SNP combinations in relation to pain sensitivity

Due to the low number of individuals homozygous for the SNP combination, both homozygous and heterozygous individuals were merged into one group which was contrasted to non-carriers.

There were no differences in PPTs, bodily pain score or coital VAS pain between carriers or non-carriers of the defined SNP combination among patients and controls analyzed separately, or in the total sample analyzed together.

### Interaction effect of GCH1-polymorphism and use of HCs on pain sensitivity

**Figure 1:**
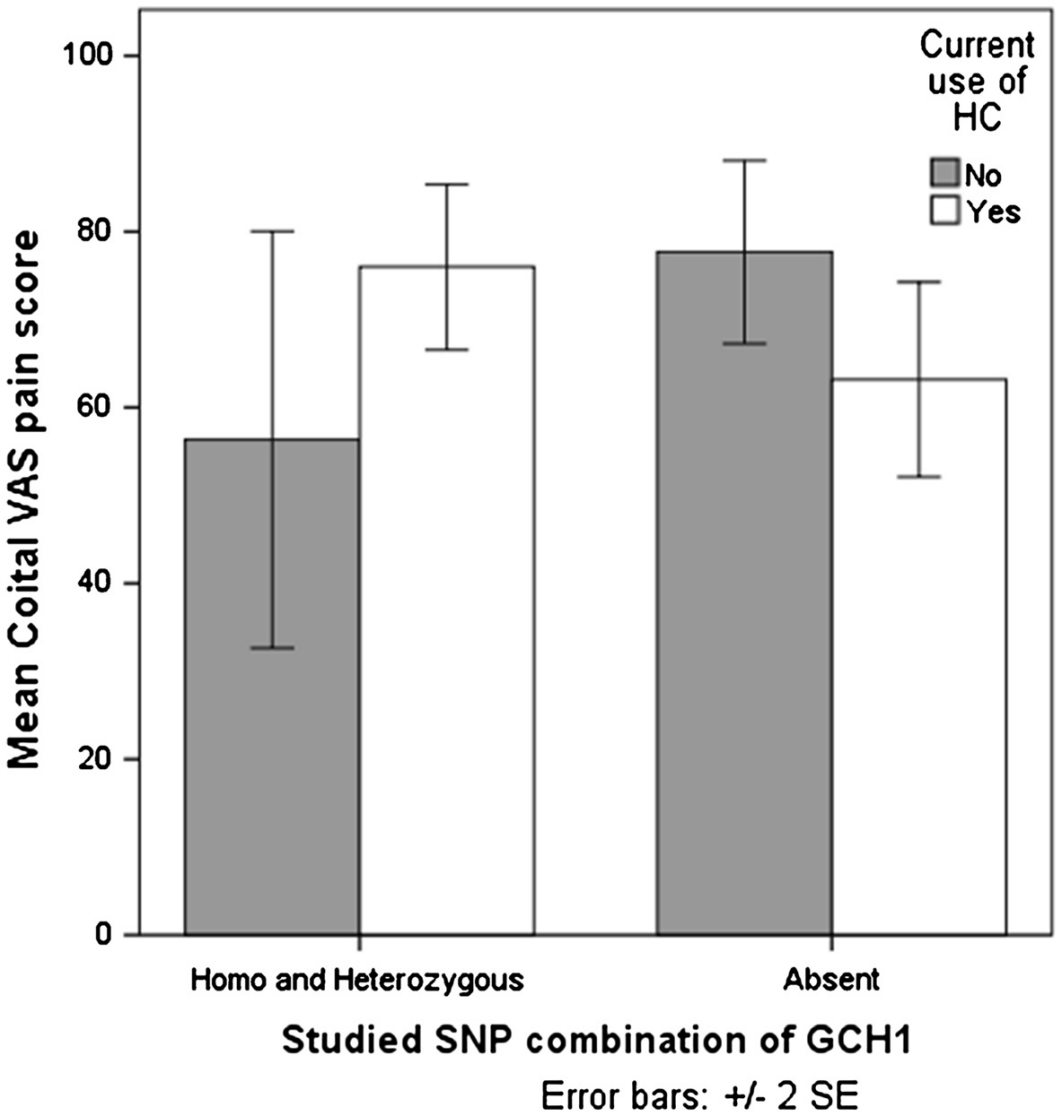
**Interaction effect of the studied SNP combination of *GCH1 *and use of hormonal contraceptives (HCs) on coital pain among patients currently receiving treatment (n = 38).** VAS = visual analogue scale.

Among all patients analyzed together there was a trend for an association between the specified SNP combination of *GCH1* and use of HCs in relation to coital VAS pain score (p < 0.07) with a low explained variance. However, when patients with current treatment were analyzed separately, there were significant main effects of the *GCH1-*gene variants, as well as significant interaction effect with HC use. The combined effect of *GCH1*-SNP combination and HC use explained approximately 8% of the variance in reported coital VAS pain, see Table 4. Among patients with current treatment not using HCs (n = 23) carriers of the specified *GCH1*-SNP combination reported lower coital VAS pain compared to non-carriers. On the other hand, in the group using HCs (n = 15), carriers of the SNP combination reported higher coital VAS pain, as shown in Figure 1.

**Table 4:** **Linear regression analyses of association between*GCH1*-SNP combination and hormonal contraceptives use among patients with current treatment (n = 36)**

	**Coital VAS pain score**		
	**df**	**F**	**p**
***GCH1*-SNP combination**	1	4.71	.037*
**Hormonal contraceptives use**	1	.11	.726
*GCH1*- SNP combination *hormonal contraceptives use	1	4.69	.038*
	Adj R^2^ = 0.084		
	**PPT arm**		
	df	F	p
***GCH1*- SNP combination**	1	4.64	.039*
**Hormonal contraceptives use**	1	1.49	.233
*GCH1*- SNP combination *hormonal contraceptives use	1	4.632	.039*
	Adj R^2^ = 0.054		
	**PPT leg**		
	df	F	p
***GCH1*- SNP combination**	1	3.92	.056
**Hormonal contraceptives use**	1	2.21	.147
*GCH1*- SNP combination *hormonal contraceptives use	1	3.90	.057
	Adj R^2^ = 0.058		

SNP = single nucleotide polymorphism, VAS = visual analogue scale, PPT = pressure pain threshold.

To further explore the association between the SNP combination and HC use in relation to the other measures of pain a series of general linear regression models (GLMás) were performed, including PPTs on the arm, leg, in vestibular area A and B and bodily pain score. In the total sample of both patients and controls together we found no association, nor in the patient group as a whole. However, separate analysis of patients with current treatment showed a relation between the *GCH1*-SNP combination, use of HCs and PPTs on the arm, and a borderline significant relation to PPTs on the leg.

**Figure 2:**
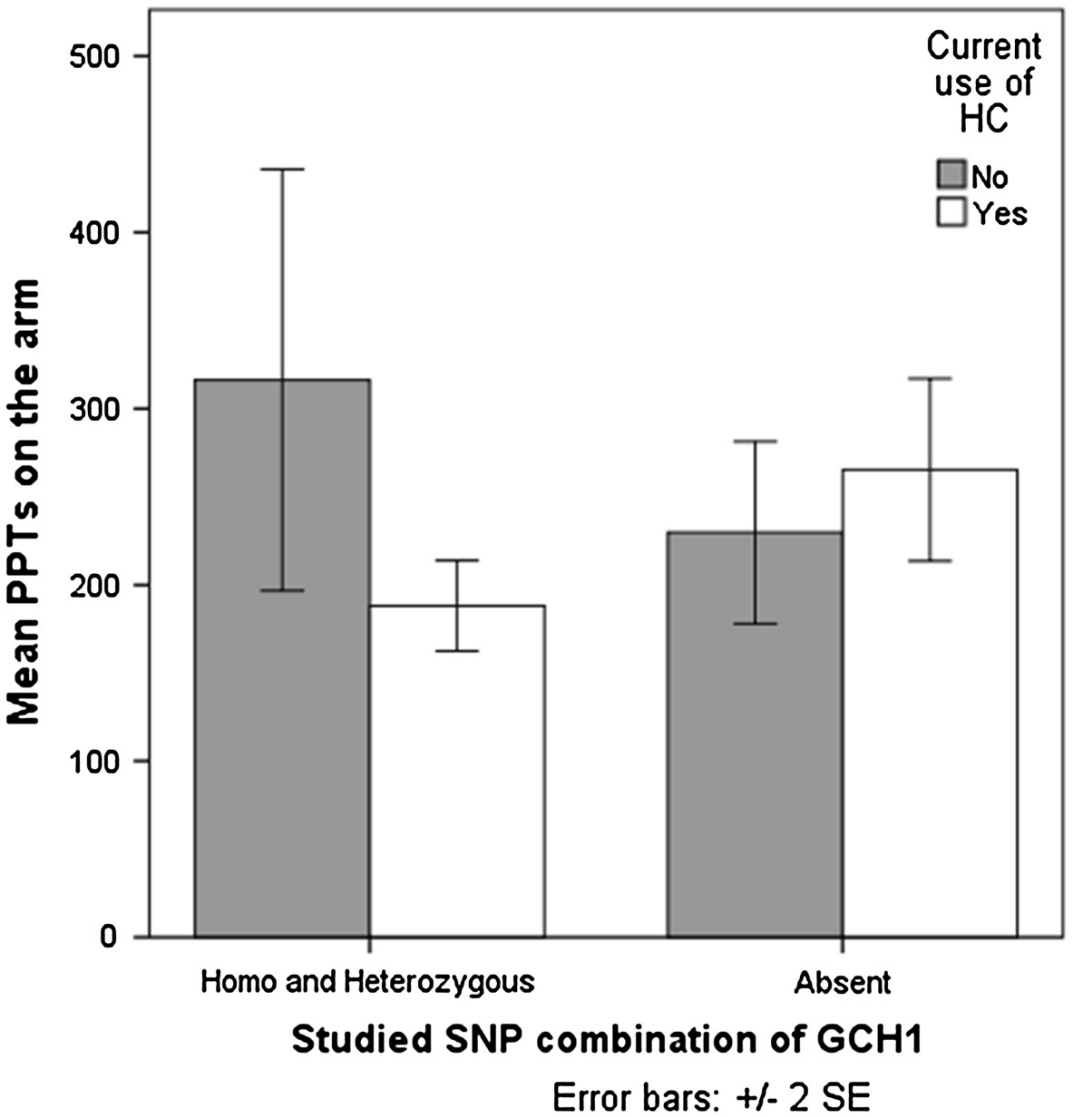
**Interaction effect of the studied SNP combination of *GCH1 *and use of hormonal contraceptives (HCs) on pressure pain thresholds (PPTs) on the arm among patients currently receiving treatment (n = 38).**

The relation between the *GCH1*-SNP combination, use of HCs and PPTs on the arm is shown in Figure 2. Among patients with current treatment not using HCs (n = 23), carriers of the specified *GCH1*-SNP combination reported lower pain sensitivity (higher PPTs) on the arm compared to non-carriers. Among patients using HCs (n = 15) the picture is inversed with higher pain sensitivity (lower PPTs) among carriers of the SNP combination.

## Discussion

The pain mechanisms involved in PVD remain an enigma. We hypothesized that the SNP combination of *GCH1* previously defined as pain protective would be less frequent among PVD patients compared to healthy controls, thus possibly contributing to the etiology of the condition. However, we found the same carrier-frequency of the different SNP combinations in patients and controls. There are several possible explanations to this lack of association. First of all pain is a very complex sensation influenced by many factors, in this case biomedical as well as psychological and sexual. The influence of a single gene polymorphism is therefore expected to be modest and difficult to statistically establish. Another possibility is that the observed pain in PVD is regulated by other aspects of the endogenous pain modulation not involving the BH4-pathway.

The studied *GCH1*-SNP combination has previously been associated with protection from the development of chronic pain after surgery for lumbar disc hernia and degeneration [26,30] but not after surgical removal of molar teeth [31] or with a diagnosis of chronic wide spread pain [34]. Experimentally, mechanical and thermal pain protective effects of the SNP combination have been found when measuring PPTs after induced hyperalgesia (through freezing or applying capsaicin) of the skin [32,35]. In our study, we found no correlation between the SNP combination and pressure pain sensitivity on non-sensitized skin on the arm or leg. Nor did we find any differences in pain measurements between patients carrying or not carrying the SNP combination in the sensitized vestibular mucosa [10-14]. The most robust associations between *GCH1* and pain responses have been appearing in acute inflammatory pain models [32,35]. Our findings are in line with the previously reported lack of association of GCH1-variation with chronic widespread pain, a predominantly female, longstanding pain condition which shares over-lapping features with PVD [34]. This might suggest a modality specific effect of *GCH1*-variation on pain with less impact on chronic pain disorders.

PVD differs from many other chronic pain disorders since the patients are pain free in the absence of provocation and the allodynia is restrained to a very limited body area. However, our results verify previous findings of higher general pain sensitivity among women with PVD compared to controls, with both lower PPTs on the arm and leg and a higher prevalence of other pain disorders (i.e. higher bodily pain score) [15-18].

In this study there were no differences in pain measurement between patients using or not using HCs. In studies investigating the effect of HCs on pain sensitivity, the results have been inconsistent. Several studies have failed to detect any differences in pain thresholds or diffuse noxious inhibitory control (DNIC) -responses between users and non-users of combined oral contraceptives (COCs) [17,40,41]. Although, in a recent study lower DNIC-responses in healthy COC users compared to non-users in low estrogen phase were found, indicating less effective endogenous pain modulation in COC-users but with only a weak correlation to endogenous estrogen levels [42]. Moreover, an altered pain sensitivity of the vestibular mucosa with decreased mechanical pain thresholds among healthy COC-users has been reported [43]. In our study, participants were examined during follicular phase, day 3–13, when endogenous estrogen levels are initially low but rising. The majority of participants with HCs used a combined pill containing both estrogen and various gestagens. In women with COCs the endogenous estrogen levels are constantly low. However, no measurements of sex hormone levels were performed in this study.

The role of gene-environment interaction has gained interest in recent years. This occurs when an environmental effect is dependent on a person’s genotype or vice versa. A limited number of studies focusing on gene-environment interaction on pain sensitivity exist. For example, desmopressin analgesia was shown to result from a three-way interaction between arginine vasopressor receptor gene variant (AVPR1A), sex and level of stress [44]. Several of the factors considered to contribute to the etiology of PVD, such as recurrent Candida infections and hormonal treatment, might have an interactive effect with genetic variation. In order to investigate one such non-genetic factor possibly interacting with GCH1-variation among women with PVD we analyzed the interactive effect of *GCH1*-polymorphism and HC use in relation to coital pain. Patients with current treatment reported higher coital VAS pain score as compared to patients with completed treatment and therefore it was anticipated that an association would be more evident in this group. We therefore analyzed patients with current treatment as an entity of its own and in this group we did find a correlation between the studied SNP combination and lower pain scores in patients not using HCs. Interestingly, in patients using HCs the relationship was inversed with higher pain sensitivity among carriers of the SNP combination. In the clinic, a subgroup of women with PVD are improved or even cured when hormonal contraceptive use is terminated [7]. The explanation to this observation is not known but it is inviting to speculate that it may result from the influence of genetic differences on endogenous pain modulation. According to our findings it is possible that PVD patients carrying the studied SNP combination would benefit the most from this intervention. However, it has been shown that HCs may have a direct effect on the vestibular mucosa [8,43] and it is not clear whether the higher coital pain ratings seen among HC users is caused by morphological changes or by hormonal effects on endogenous pain modulation or an interaction of both. Possibly the mucosal effects of HCs are greater than the pain modulatory effect which could explain the fact that among users of HCs there seem to be no pain protective effect of the studied *GCH1*-SNP combination. We also found a relation between the *GCH1*-SNP combination, use of HCs and PPTs on the arm which strengthens the idea of an interactive effect of these variables.

Based on our findings of a GCH1-gene by HC interaction in relation to pain sensitivity, it can be speculated that there would be a gene by sex interaction as well as a gene by sex-steroids interaction. Studies have shown a gene-sex interaction with pain sensitivity regarding genes involved in the opioid and desmopressin systems [44,45]. To our knowledge, this has not been studied regarding GCH1. Most previous studies have participants of mixed sexes and the results have not been analyzed separately. Furthermore, a frequency of the studied SNP combination of approximately 15% has been reported in a normal Caucasian population of mixed sexes [28,29]. In this material of young Swedish women we found a carrier-frequency of the SNP combination (homo- and heterozygous), among patients and controls, of approximately 28%, this in line with findings by Dabo and colleagues [36].

The exact role of this gene in human endogenous pain modulation still needs to be clarified and genetic polymorphism in other genes possibly involved in pain modulation in PVD remains a subject for future research.

In the study design 100 participants in each group were estimated to give sufficient power for this study based on a method to optimize sample size in candidate gene studies described by [Belfer et al. 46]. Although an even larger study group would have strengthen the results, our study population is well defined with reliable inclusion criteria and there is strong evidence to support the role of the *GCH1*-gene in human endogenous pain modulation. Furthermore, by analyzing the gene polymorphism in correlation to continuous variables such as pain measurements the power is increased. Regarding the use of hormonal contraceptives one might argue that a more homogenous group with respect to the hormonal content would have been preferable, since in our material the participants used both combined and progestogen only methods. However a large variety of HCs are commonly used and inclusion of participants in this study was based on the PVD diagnosis solely.

## Conclusion

The results of this study gave no support to the hypothesis that polymorphism in the *GCH1*-gene contributes to the etiology of PVD. However, among patients currently receiving treatment an interaction effect of the SNP combination of *GCH1*, previously identified as pain-protective, and use of hormonal contraceptives on pain sensitivity was found. These findings offer a possible explanation to the clinically known fact that some PVD patients improve after cessation of hormonal contraceptives, indicating that PVD patients carrying the studied *GCH1-*SNP combination would benefit from this intervention.

## Methods

Ninety-eight women with PVD were recruited. The inclusion criteria for patients were: age ≥ 18 years, PVD defined as pain at vestibular contact and vaginal entry, with duration of symptoms of ≥6 months based on the initial exam at the time of diagnosis. The exclusion criteria were: local infection or dermatological causes to dyspareunia, major psychiatric or medical disease and pregnancy. Controls consisted of 102 healthy women, mostly medical students and hospital staff. The inclusion criteria were age > 18 years, regular menstruation. Exclusion criteria were: dyspareunia, major medical or psychiatric disease, use of regular analgesics or anti-depressants and pregnancy. All participants received oral and written information about the study and provided informed consent and the study was approved by the local ethical committee.

### Clinical data

All participants were invited to a single testing session carried out on days 3–13 of the menstrual cycle. A comprehensive questionnaire regarding psychosocial, medical and gynecological history was filled out. Other frequent bodily pain disorders were reported. The number of bodily pain disorders was used to create a bodily pain score for each participant with a range from 0 – 5. Patients completed an additional questionnaire including questions related to their PVD. For patients engaging in vaginal intercourse, the intensity of coital pain during the last month was scored on a Visual Analogue Scale (VAS) with a range from 0 –100, where 0 represents no pain and 100 represents the worst pain imaginable. Pressure pain thresholds (PPTs) on the arm and leg was measured using a pressure algometer (Somedic Sales AB, Hörby, Sweden) with a disc shaped rubber top of 1 cm^2^. The arm was first tested, on the deltoid muscle, 3 cm proximal to the tendon insertion of the muscle. Subsequently the leg was tested, on the anterior tibial muscle, approximately 5 cm below and 3 cm lateral to the tibial tuberosity. Testing was performed on the opposite side of the subjects reported dominant hand. The device was applied perpendicularly to the skin and the pressure was increased by 50–75 kPa/s. The participants were asked to report the PPT, defined as when the sensation changed from discomfort to pain by pushing a button. The pressure, displayed digitally, was then registered. The measurement was repeated twice and the mean value was registered. All participants were given a careful explanation of the procedure and a training session on the opposite arm before the testing started. Measurements were carried out by the same examiner who to this point was blinded to whether the participant belonged to the patient or the control group as well as to the participant’s genotype.

At this stage, the patient or control status was revealed; PPTs in the vestibular mucosa were measured in patients only, using vulvar algesiometers [47]. The algesiometers consisted of cylindrical devices containing metal springs of varying compression rates with a cotton swab top. The set exerts a pressure ranging from 3–1000 g. Two different areas of the vestibule was tested, area A, in the anterior vestibule, close to the urethra and area B, in the posterior vestibule, close to the opening of the Bartholinás glands, both on the right side of the vaginal opening. The pressure was slowly and successively increased until the participator orally reported the PPT. The measurement was repeated twice and the mean value was used for analysis. All subjects were given a careful explanation of the procedure before the testing started.

### DNA isolation

Venous blood samples were collected in tubes containing EDTA and the blood samples were stored at −70° until further processing. The Magtration 12GC system (Precision System Science, Chiba, Japan) and the Magazorb® DNA Common Kit-200 (PSS, Chiba, Japan) were used for preparation of the total genomic DNA. From each sample 200 μl whole blood was used and the final volume of the DNA extract was 100 μl. The concentration of the DNA was determined with Nanodrop Spectrophotometer (Nanodrop Techncologies Inc., Wilmington, DE, USA).

### Genotyping of the SNPs in the GCH1

We focused on three SNPs to define the pain-protective SNP combination of *GCH1*; *dbSNP rs8007267G > A (c.-9610 G > A), dbSNP rs3783641A > T (c343 + 8900A > T)* and dbSNP rs10483639C > G (c.*4279 > G). For the analysis of these three SNP: s, we applied the TaqMan SNP genotyping assay (Applied Biosystems, Foster City, USA). Briefly, Applied Biosystems designed the primers and the allele-specific probes. The assay included target-specific PCR primers and TaqMan MGB probes labeled with two special dyes, FAM and VIC. Genomic DNA (5 ng), water, TaqMan Universal PCR master mix and TaqMan genotyping assay mix was added to each well in a 384-well plate, in a total volume of 5ul. The genotyping was carried out, using the ABI7900HT genetic detection system (Applied Biosystems, Foster City, USA) according to the manufacturersá instructions, with the following amplification protocol: 10 min at 95°C and 40 cycles of 15 s at 92°C and 1 min at 60°C.

### Statistics

The Statistica program (version 10, StatSoft Inc., Tulsa, OK, USA) and the Statistical package for the Social Sciences program (version 20, SPSS Inc., Chicago, IL, USA) were used. In comparisons of pain sensitivity between groups the analysis of variance (ANOVA)-test was used. For ordinal data the non parametric equivalents Kruskal-Wallis and the Mann–Whitney *U*-test were used respectively and for SNP frequencies the Chi^2^-test. To analyze a possible interaction between *GCH1-*SNP combination and use of HCs in relation to pain sensitivity we used general linear regression models (GLM) with main effects of *GCH1*-SNP combination and use of HCs as well as interaction effects of *GCH1-*SNP combination and HCs together. A significance level of p < 0.05 was used for all statistical tests and a confidence interval of 95% for the logistic regression analyses.

## Abbreviations

PVD: Provoked vestibulodynia (former vulvar vestibulitis); SNP: Single nucleotide polymorphism; HWE: Hardy-Weinberg equilibrium; GCH1: Guanosine triphosphate cyklohydrolase 1; BH4: 6(R)-L-erythro-5,6,7,8-tetrahydrobiopterin; COC: Combined hormonal contraceptives; HC: Hormonal contraceptives; PPT: Pressure pain threshold; VAS: Visual analogue scale; DNIC: Diffuse noxious inhibitory control; GLM: General linear regression models.

## Competing interests

The authors declare that they have no competing interests.

## Authors’ contributions

UH participated in the design of the study, collected the data, participated in statistical analysis and drafted the manuscript. NBS conceived of the study, participated in its design and coordination and helped to draft and the manuscript. AG carried out the SNP analyses and contributed to the drafting of the manuscript. FN participated in the study design and revised the manuscript. KN performed the statistical analysis and contributed to the interpretation of data and drafting of the manuscript, tables and figures. UJ participated in the design of the study and revised the manuscript. All authors read and approved the final manuscript.

## Author details

^1^Department of Clinical Sciences, Danderyd Hospital, Karolinska Institutet, Division of Obstetrics and Gynecology, Stockholm, Sweden. ^2^Department of Pharmaceutical Biosciences, Division of Biological Research on Drug Dependence, Uppsala University, Uppsala, Sweden. ^3^Center for clinical research, Uppsala University, County Council of Västmanland Central Hospital, Västerås, Sweden.
